# A Global Systematic Review and Meta‐Analysis of *Giardia duodenalis* in Rabbits: Epidemiology, Genetic Diversity and Possible Zoonotic Concerns

**DOI:** 10.1002/vms3.70176

**Published:** 2024-12-18

**Authors:** Ali Asghari, Mohammad Reza Mohammadi, Leila Naseri, Laya Shamsi, Milad Badri, Ali Pouryousef

**Affiliations:** ^1^ Medical Microbiology Research Center Qazvin University of Medical Sciences Qazvin Iran; ^2^ Department of Bacteriology, Faculty of Medical Sciences Tarbiat Modares University Tehran Iran; ^3^ Department of Anatomical Sciences, School of Medicine Ilam University of Medical Sciences Ilam Iran; ^4^ Clinical Research Development Unit, Shahid Mostafa Khomeini Hospital Ilam University of Medical Sciences Ilam Iran; ^5^ Department of Pathobiology, Faculty of Veterinary Medicine Urmia University Urmia Iran; ^6^ Leishmaniasis Research Center Sabzevar University of Medical Sciences Sabzevar Iran

**Keywords:** assemblage, epidemiology, *Giardia duodenalis*, meta‐analysis, rabbits, systematic review

## Abstract

**Background:**

*Giardia duodenalis* is a pathogenic protozoan responsible for gastrointestinal infections in various hosts, including rabbits. Understanding its epidemiology, genetic diversity and zoonotic implications is vital for public health and veterinary medicine. This systematic review and meta‐analysis aimed to synthesize global data on the prevalence of *G. duodenalis* in rabbit populations, assess the genetic diversity of the isolates and evaluate the associated zoonotic potential.

**Methods:**

We conducted a thorough literature search across multiple databases (PubMed, Scopus, Web of Science and Google Scholar) for studies published until 18 October 2024. Only peer‐reviewed articles reporting original research on *G. duodenalis* in rabbits were included. We extracted data on prevalence rates, testing times, publication years, countries, continents, WHO regions, diagnostic methods, genes used, assemblages and sub‐assemblages. A meta‐analysis using random‐effects models was performed to calculate pooled prevalence rates, with the *I* ^2^ index used to assess heterogeneity.

**Results:**

A total of 26 studies/datasets were analysed, covering data from 5543 rabbits across 15 countries. The estimated pooled prevalence of *G. duodenalis* in rabbits was 12.1% (95% CI: 7%–20%), with substantial heterogeneity (*I* ^2^ = 96.5%). Geographic analysis showed higher prevalence rates in Africa and the AFR WHO region (72.3%, 95% CI: 61.7%–80.8%). Genetic analysis revealed three zoonotic assemblages (A, B and E) and two zoonotic sub‐assemblages (AI and BIV) of *G. duodenalis* in rabbits, highlighting concerns over zoonotic transmission.

**Conclusions:**

The findings highlight the global presence and genetic diversity of *G. duodenalis* in rabbits, indicating potential zoonotic risks. Ongoing monitoring and research are essential to clarify the transmission dynamics and public health implications of *G. duodenalis* in these animals. Raising awareness among pet owners, veterinarians and public health officials is vital to mitigate potential zoonosis.

## Graphical abstract

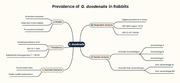

## 1 Introduction

*Giardia duodenalis* (also known as *Giardia intestinalis* and *Giardia lamblia*) is a common zoonotic protozoan parasite and one of the most widespread gastrointestinal pathogens, causing giardiasis in humans and various animal species (J. Li et al. 2017). This protozoan parasite causes approximately 280 million cases of giardiasis each year, leading to diarrhoea and other intestinal symptoms, with asymptomatic infections also common (Einarsson, Ma'ayeh, and Svärd 2016). Acknowledging its significance, the World Health Organization (WHO) categorized giardiasis as a neglected disease in 2004 (Savioli, Smith, and Thompson 2006).

*G. duodenalis* cysts, the infectious stage, are excreted in faeces and spread via the faecal‐oral route (Adam 2021). They can survive in water and other environments, even against chlorine disinfectants, contributing to many waterborne disease outbreaks (Castro‐Hermida et al. 2008). In the last 40 years, at least 132 documented giardiasis outbreaks have been waterborne (Carmena 2010). Foodborne outbreaks have also occurred due to contaminated food handled by infected workers, ranking *G. duodenalis* 11th among the 24 foodborne parasite species identified by the Food and Agriculture Organization (FAO) of the United Nations (Dixon 2021; Barlaam et al. 2022). Although *G. duodenalis* is a global concern due to its outbreak potential, treatment options are limited, and there are no approved vaccines. Effectively managing *G. duodenalis* infections necessitates a comprehensive understanding of infection sources and transmission dynamics (Ansell et al. 2015; Sangkanu et al. 2022).

Molecular diagnostics for *G. duodenalis* infections mark a significant advancement in understanding the epidemiology of this parasite. Molecular typing tools targeting specific genes such as small subunit ribosomal RNA (*SSU rRNA*), glutamate dehydrogenase (*gdh*), triosephosphate isomerase (*tpi*) and β‐giardin (*bg*) are essential for identifying *G. duodenalis* genotypes/assemblages (Cacciò et al. 2008; Wang et al. 2014; Rafiei et al. 2020). *G. duodenalis* comprises genetically diverse species grouped into assemblages A–H. Assemblages A and B have zoonotic potential and are linked to diarrhoea in humans and animals, while C–H are specific to certain animal hosts (Heyworth 2016; Chourabi et al. 2021; Seabolt, Roellig, and Konstantinidis 2022). Four sub‐assemblages (AI–AIV and BI–BIV) were identified by an allozyme study within assemblages A and B; the majority of these sub‐assemblages (AI, AII, BIII and BIV) have been mainly found in humans (Cacciò et al. 2008; Feng and Xiao 2011; Zahedi et al. 2020).

In recent years, the acknowledgement of wildlife and domestic animals, particularly rabbits, as potential reservoirs of zoonotic pathogens have gained considerable attention (Pacha et al. 1987; Chilvers et al. 1998; Sulaiman et al. 2003; Lebbad et al. 2010; Beck, Sprong, Bata, et al. 2011; Beck, Sprong, Lucinger, et al. 2011; W. Zhang et al. 2012; X. Zhang et al. 2018; Nolan et al. 2013; Rewatkar et al. 2013; Liu et al. 2014; Pantchev et al. 2014; Qi et al. 2015; Mosallanejad, Avizeh, and Razi Jalali 2016; Koehler et al. 2016; Akinkuotu et al. 2018; Marhoon, Mattar, and Mohammad 2018; Sarzosa et al. 2018; Jiang et al. 2018; Kurnosova, Arisov, and Odoyevskaya 2019; T.‐S. Li et al. 2020; Zahedi et al. 2020; Tang et al. 2021; ElBakri et al. 2021; Baptista et al. 2023; Rego et al. 2023). As rabbits are commonly kept as pets, livestock and laboratory animals, they may significantly impact the epidemiology of *G. duodenalis* and its transmission to humans (González‐Redondo and Contreras‐Chacón 2012). Despite the growing body of literature focusing on *G. duodenalis* in different animal hosts, there remains a dearth of comprehensive studies specifically examining its prevalence in rabbit populations. Therefore, a systematic review and meta‐analysis of available studies are warranted to elucidate the epidemiology of *G. duodenalis* in rabbits, characterize its genetic diversity and explore the possible zoonotic concerns associated with this parasite. This review aimed to synthesize existing data, quantify the prevalence of *G. duodenalis* in rabbits globally and provide insights into its genetic variability and implications for public health.

## 2 Methods

### 2.1 Study Reporting

This systematic review and meta‐analysis was conducted following the Preferred Reporting Items for Systematic Reviews and Meta‐Analyses (PRISMA) guidelines (Moher et al. 2015).

### 2.2 Research Questions

The primary research questions addressed in this review included: What is the global prevalence of *G. duodenalis* in rabbit populations? What are the assemblages and sub‐assemblages among *G. duodenalis* isolates from rabbits? Are there potential zoonotic implications associated with *G. duodenalis* isolates found in rabbits?

### 2.3 Search Strategy

Comprehensive searches were conducted in the following electronic databases: PubMed, Web of Science, Scopus and Google Scholar. The search strategy included terms related to ‘*Giardia duodenalis*’, ‘Rabbits’, ‘Epidemiology’, ‘Genetic diversity’, ‘Assemblage’, ‘Sub‐assemblage’ and ‘Zoonotic’. Boolean operators (AND, OR) were used to combine keywords effectively. The search was limited to articles published up to 18 October 2024, and only studies published in English were included.

### 2.4 Eligibility Criteria

Studies were included in the review if they reported the prevalence of *G. duodenalis* in domestic or wild rabbits through molecular, serological or microscopic investigations, specifying both total and infected sample sizes. Exclusion criteria included studies lacking sufficient epidemiological data, as well as reviews, commentaries or opinion pieces without primary data; animal studies unrelated to rabbits; human studies and experimental reports.

### 2.5 Study Selection

Two independent reviewers (A.A. and A.P.) screened the titles and abstracts of all identified articles for eligibility. The full texts of potentially eligible studies were then assessed by other authors (M.R.M., L.N., L.S. and M.B.). Disagreements were resolved through discussion, with a lead reviewer (A.A.) consulted as needed.

### 2.6 Data Extraction and Management

Data were extracted using a pre‐designed form capturing study characteristics (authors, publication year, geographical location), sample size, common and scientific names of rabbits, *G. duodenalis* prevalence rates, isolated assemblages/sub‐assemblages, diagnostic methods and genotyping/identification genes. Two reviewers independently completed the data extraction, resolving discrepancies by consensus.

### 2.7 Quality Assessment

The Joanna Briggs Institute (JBI) checklist for reporting prevalence data in systematic reviews and meta‐analyses was used for quality assessment, covering key criteria such as sample size, participant descriptions, data analyses, reliable objectives, appropriate statistical methods, confounding factors and subgroups (Munn et al. 2014). Articles scoring ≤ 3 were excluded; those scoring 4–6 and ≥ 7 were classified as medium and high quality, respectively.

### 2.8 Software and Statistical Analysis

Statistical analyses were performed using comprehensive meta‐analysis (CMA) v3 software. A *p*‐value of < 0.05 was considered statistically significant throughout the analysis. Prevalence data were pooled using a random‐effects model to account for heterogeneity among studies. The *I* ^2^ statistic was calculated to quantify the variability; *I* ^2^ values of 25%, 50% and 75% were interpreted as low, moderate and high heterogeneity, respectively. Sub‐group analyses were performed based on publication year, country, continent, WHO regions and sample size. The genetic diversity of *G. duodenalis* was analysed descriptively, summarizing different assemblages/sub‐assemblages and their potential implications for zoonotic transmission.

### 2.9 Sensitivity Analysis

A sensitivity analysis was conducted to evaluate the robustness of the results. This involved re‐evaluating the meta‐analysis excluding single datasets/studies and assessing the impact on overall prevalence estimates.

## 3 Results

### 3.1 Study Selection

**FIGURE 1:**
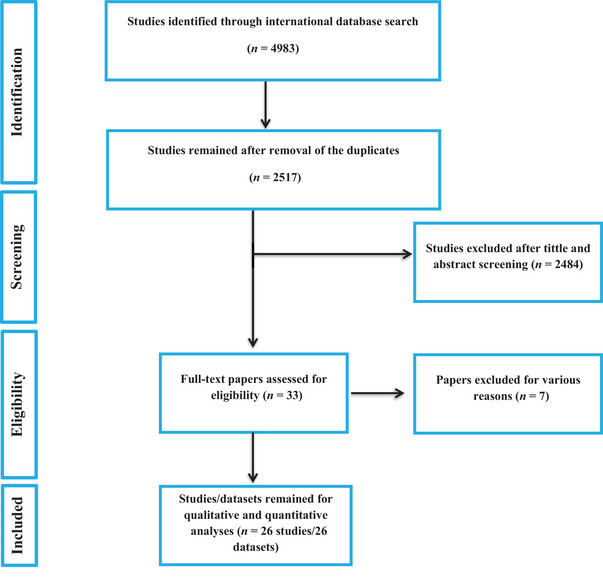
Flowchart depicting the process of included studies in the present review.

The literature search identified 4983 articles across all databases. After removing duplicates, 2517 titles and abstracts were screened for eligibility. Of these, 33 studies were deemed potentially relevant and underwent full‐text and quality assessment. Ultimately, 26 studies met the eligibility criteria and were included in this systematic review and meta‐analysis. Figure 1 shows a PRISMA flow diagram detailing the study selection process.

### 3.2 Study Characteristics

Table 1 summarizes the characteristics of the included studies, which varied in sample size (1–995), geographical locations, and diagnostic methodologies. Studies with a sample size of one (two papers) were excluded from the statistical analysis. In total, 5543 rabbits were included from 26 studies, with prevalence rates reported across 15 countries on six continents and six WHO regions. Most studies (19 papers) used molecular techniques to detect *G. duodenalis*, while serological and microscopic methods were employed in one and six studies, respectively. Among the 19 molecular studies, 17 reported *G. duodenalis* assemblages and 6 reported sub‐assemblages.

**TABLE 1:** Key characteristics of 26 articles/datasets on the prevalence and distribution of *G. duodenalis* assemblages/sub‐assemblages in rabbits.

References	Host's common name	Host's scientific name	Time tested	Country	Total no.	Infected no.	Prevalence (%)	Method	Tested gene	Assemblages	Sub‐assemblages
Pacha et al. (1987)	Rabbit	—	1983–1985	USA	15	0	0	MIC^c^	—	—	—
Chilvers et al. (1998)	European rabbit	*Oryctolagus cuniculus*	1991–1994	New Zealand	5	1	20	MIC	—	—	—
Sulaiman et al. (2003)	Rabbit	—	2000	—	1	1	100	MOL^d^	MLG^f^	B	—
Lebbad et al. (2010)	Rabbit	—	2002–2008	USA	1	1	100	MOL	MLG	B	—
Beck, Sprong, Bata, et al. (2011)	European hare	*Lepus europeus*	2006–2009	Sweden	73	0	0	MIC	—	—	—
Beck, Sprong, Lucinger, et al. (2011)	European rabbit	*Oryctolagus cuniculus*	2005	Croatia	2	0	0	MOL	MLG	—	—
W. Zhang et al. (2012)	Rabbit	—	2008–2010	Croatia	378	28	7.4	MOL	TPI^g^	B	—
Nolan et al. (2013)	European rabbit	*Oryctolagus cuniculus*	2009–2011	China	263	3	1.1	MOL	MLG	A	—
Rewatkar et al. (2013)	Rabbit	—	2008	Australia	42	8	19	MIC	—	—	—
Liu et al. (2014)	Rabbit	—	2008–2011	India	14	14	100	MOL	MLG	B	—
Pantchev et al. (2014)	Rabbit	—	2006–2012	China	528	40	7.6	MOL	MLG	B	—
Qi et al. (2015)	Rabbit	—	2008–2011	Germany^b^	955	80	8.4	MOL	MLG	B, E	BIV
Koehler et al. (2016)	European rabbit	*Oryctolagus cuniculus*	2011–2015	China	97	1	1	MOL	TPI	A	AI
Mosallanejad, Avizeh, Razi Jalali (2017)	Rabbit	—	2011–2014	Australia	58	5	8.6	SER^e^	—	—	—
Akinkuotu et al. (2018)	European rabbit	*Oryctolagus cuniculus*	2016	Iran	83	60	72.3	MOL	MLG	B	BIV
Jiang et al. (2018)	European rabbit	*Oryctolagus cuniculus*	2014	Nigeria	426	42	98.6	MOL	MLG	B	—
Marhoon, Mattar, and Mohammad (2018)	European rabbit	*Oryctolagus Cuniculus*	2016–2017	China	55	9	16.3	MIC	—	—	—
Sarzosa et al. (2018)	Rabbit	—	2014–2016	Iraq	39	6	15	MOL	TPI	B	—
X. Zhang et al. (2018)	Rabbit	—	UC^a^	Ecuador	321	6	1.9	MOL	MLG	B	BIV
Kurnosova, Arisov, and Odoyevskaya (2019)	European rabbit	*Oryctolagus cuniculus*	UC	China	165	3	1.8	MIC	—	—	—
T.‐S. Li et al. (2020)	Long‐haired rabbit, New Zealand white rabbit, Tolai	—	UC	Russia	616	69	11.2	MOL	MLG	A, B, E	—
Zahedi et al. (2020)	Hare	—	2013–2015	China	217	16	7.4	MOL	MLG	B	BIV
Elbakri et al. (2021)	Rabbit	*Oryctolagus* spp.	UC	Australia	2	2	100	MOL	MLG	—	—
Tang et al. (2021)	European rabbit	—	2017–2018	UAE	537	19	3.7	MOL	MLG	B, E	—
Baptista et al. (2023)	Rex rabbit, IRA rabbit Domestic rabbit	*Oryctolagus cuniculus domesticus*	2020	China Brazil	100	40	40	MOL	MLG	A	—
Rego et al. (2023)	European rabbit, Granada hare	*Oryctolagus cuniculus, Lepus granatensis*	2012–2021	Spain	550	153	27.8	MOL	—	B	BIV

^a^ Unclear.

^b^ This article gathered samples from several European countries, including Germany.

^c^ Microscopic detection.

^d^ Molecular detection.

^e^ Serological detection.

^f^ Multilocus genotyping with more than one gene.

^g^ Triosephosphate isomerase gene.

### 3.3 Global Prevalence of *G. duodenalis* in Rabbits

**FIGURE 2:**
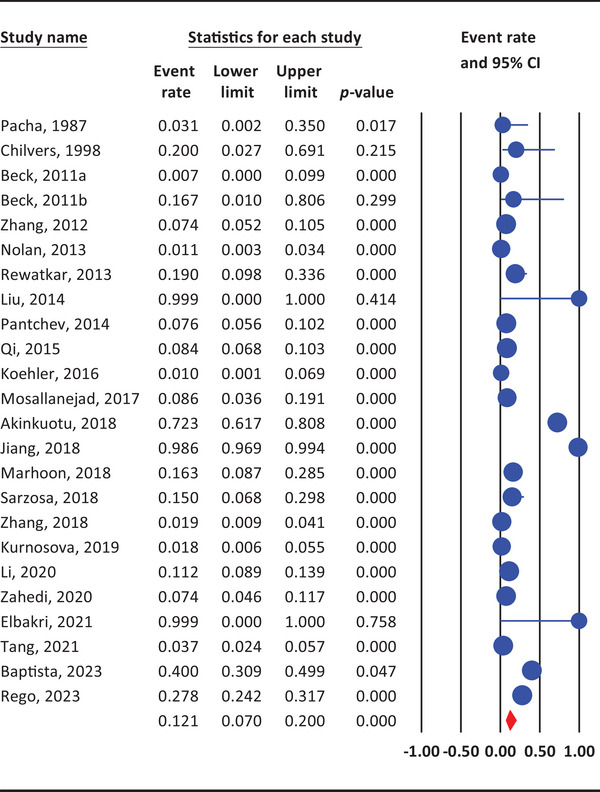
The weighted prevalence of *G. duodenalis* in rabbits, based on data from the included studies, using a random‐effects model and 95% confidence intervals.

The pooled prevalence of *G. duodenalis* in rabbits was 12.1% (95% CI: 7%–20%, *I* ^2^ = 96.5%), reflecting significant heterogeneity among studies (Figure 2). Subgroup analyses showed variations in prevalence rates by publication year, country, continent, WHO region, and sample size (Table 2 and Figures S1–S5).

**TABLE 2:** Subgroup analysis of *G. duodenalis* in rabbits by publication year, continent, WHO region, country and sample size.

Subgroup variable	Prevalence % (95% CI)	Heterogeneity (Q)	*df* (Q)	*I^2^* (%)	*p*‐value
Publication year					
< 2000 2011–2014 2015–2018 2019–2023	9.9 (1.5–44) 6.5 (3.5–11.9) 20.2 (4.6–57.2) 10.7 (4.8–21.9)	1.3 23.2 400.4 174.7	1 6 7 6	20.9 74.1 98.2 96.5	*p* > 0.05 *p* < 0.05 *p* < 0.05 *p* < 0.05
Continent					
Africa Asia Europe North America Oceania South America	72.3 (61.7–80.8) 15.4 (6.9–30.9) 6.9 (2.1–20.4) 3.1 (0.2–35) 3.6 (0.9–13) 26.7 (9.1–57.1)	0 298.5 92.7 0 13.9 7.3	0 10 4 0 3 1	0 96.6 95.7 0 78.5 86.2	*p* > 0.05 *p* < 0.05 *p* < 0.05 *p* > 0.05 *p* < 0.05 *p* < 0.05
WHO region					
AFR AMR EMR EUR SEAR WPR	72.3 (61.7–80.8) 19.9 (6.1–48.8) 12.9 (7.8–20.7) 6.9 (2.1–20.4) 19 (9.8–33.6) 9.8 (4.2–21.1)	0 11 1.6 92.7 0 313.1	0 2 2 4 0 10	0 81.1 95.4 92.9 0 0	*p* > 0.05 *p* < 0.05 *p* > 0.05 *p* < 0.05 *p* > 0.05 *p* < 0.05
Country					
Australia Brazil China Croatia Ecuador Germany India Iran Iraq New Zealand Nigeria Russia Spain UAE USA	2.3 (0.5–10.6) 40 (30.9–49.9) 16.1 (5.6–38.2) 3.4 (0.1–48.9) 15 (6.8–29.8) 7.6 (5.6–10.2) 19 (9.8–33.6) 8.6 (3.6–19.1) 16.3 (8.7–28.5) 20 (2.7–69.1) 72.3 (61.7–80.8) 1.8 (0.6–5.5) 27.8 (24.2–31.7) 100 (0–100) 3.1 (0.2–35)	12.1 0 291.9 2.6 0 0 0 0 0 0 0 0 0 0 0	2 0 6 1 0 0 0 0 0 0 0 0 0 0 0	83.5 0 97.9 61.4 0 0 0 0 0 0 0 0 0 0 0	*p* < 0.05 *p* > 0.05 *p* < 0.05 *p* > 0.05 *p* > 0.05 *p* > 0.05 *p* > 0.05 *p* > 0.05 *p* > 0.05 *p* > 0.05 *p* > 0.05 *p* > 0.05 *p* > 0.05 *p* > 0.05 *p* > 0.05
Sample size					
≤ 100 > 100	15.1 (6.7–30.7) 10.1 (5–19.3)	104.9 462.5	12 10	88.5 97.8	*p* < 0.05 *p* < 0.05

### 3.4 Genetic Diversity of *G. duodenalis* in Rabbits

Genetic analysis of *G. duodenalis* isolates identified multiple assemblages. Assemblage B was the most common, appearing in 14 studies, followed by assemblage A in four and assemblage E in three. Sub‐assemblage analysis revealed one subtype in assemblage A (sub‐assemblage AI) and one in assemblage B (sub‐assemblage BIV) (Table 1).

### 3.5 Zoonotic Potential of *G. duodenalis* Isolates in Rabbits

Available classified information indicated that rodents were the source of infection for three zoonotic assemblages (A, B and E) and two zoonotic sub‐assemblages (AI and BIV) of *G. duodenalis* (Table 1).

### 3.6 Sensitivity Analysis

**FIGURE 3:**
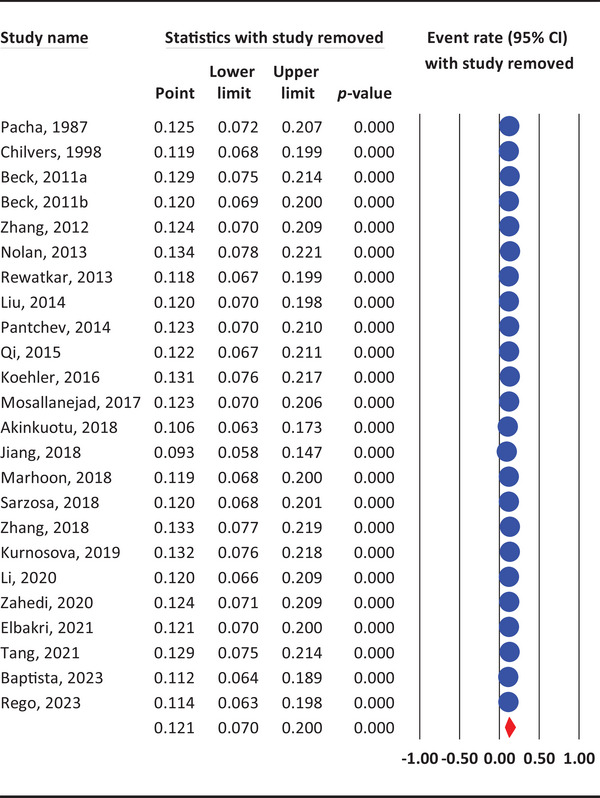
Sensitivity analysis of *G. duodenalis* prevalence in rabbits.

Sensitivity analysis indicated that the overall prevalence estimates remained stable when individual studies were removed from the meta‐analysis, suggesting robust findings. The exclusion of single datasets/studies did not significantly alter the pooled prevalence, affirming the reliability of the results (Figure 3).

### 3.7 Quality Assessment

Quality assessment using the Joanna Briggs Institute (JBI) checklist showed that 10 studies scored ≥ 7 (high quality), while 16 studies scored between 4 and 6 (medium quality) (Table S1).

## 4 Discussion

The pooled prevalence of *G. duodenalis* in rabbits was found to be 12.1%, with a wide confidence interval (95% CI: 7%–20%) and significant heterogeneity (*I* ^2^ = 96.5%). This variability could be attributed to differences in geographical regions, diagnostic methodologies, sample sizes, and the year of publication, all of which influenced the prevalence rates. No global meta‐analyses have been conducted on various parasites in rabbits, making result comparisons problematic. However, *G. duodenalis* prevalence rates in cattle (22%, 95% CI: 17%–28%) (Taghipour et al. 2022); dogs (15.2%, 95% CI: 13.8%–16.7%); cats (12%, 95% CI: 9.2%–15.3%) (Bouzid et al. 2015) and pigs (9.1%, 95% CI: 5.6%–14.3%) (Asghari et al. 2023) have been reported. Although differences in the number of studies, geographic regions, and diagnostic methods complicate comparisons, livestock like cattle and companion animals such as dogs appear to be significant sources of *G. duodenalis* infection compared to cats, pigs and rabbits. Nevertheless, rabbits with a 12.1% prevalence should not be overlooked as a potential source of *G. duodenalis* infections. Of note, most analyses in these meta‐analyses are based on limited data/studies from specific areas, which complicates result interpretation and warrants caution in drawing conclusions. Moreover, the ecological and behavioural factors influencing *G. duodenalis* transmission in rabbits are not well understood, further complicating our comprehension of their role as reservoirs. The observed prevalence rates in rabbits may differ substantially across regions. Environmental conditions, such as the presence of contaminated water sources and interactions with other infected wildlife, can greatly affect transmission dynamics.

The robustness of the prevalence estimates was affirmed through sensitivity analyses, which indicated that the overall pooled prevalence of *G. duodenalis* in rabbits remained stable despite the removal of individual studies. This reinforces the reliability of our findings and indicates that the conclusions drawn are well supported by the data. Nonetheless, the high level of heterogeneity warrants cautious interpretation of the pooled estimates and emphasizes the need for further studies that explore the factors contributing to this variability.

Due to data limitations, a clear trend between *G. duodenalis* prevalence in rabbits and the analysed subgroups cannot be established. However, based on the year of publication, the highest prevalence of *G. duodenalis* in rabbits was 20.2% (95% CI: 4.6%–57.2%) in 2018–2015, while the lowest was 6.5% (95% CI: 3.5%–11.9%) in 2011–2014. Furthermore, it is worth noting that the observed fluctuations in prevalence rates may be influenced by various factors, such as sampling methods, population dynamics and environmental conditions. For instance, variations in diagnostic techniques over the years could potentially contribute to discrepancies in reported prevalence levels. Moreover, changes in rabbit husbandry practices, habitat alterations and climatic factors may also play a significant role in infection rates. The prevalence assessment by continent indicated that, from individual studies, Africa had the highest prevalence at 72.3% (95% CI: 61.7%–80.8%), while North America had the lowest at 3.1% (95% CI: 0.2%–35%). In terms of weighted prevalence, Asia reported the highest at 15.4% (95% CI: 6.9%–30.9%, 11 studies) and Oceania the lowest at 3.6% (95% CI: 0.9%–13%, 4 studies). These findings highlight significant geographical variations in the distribution of *G. duodenalis* among rabbit populations, underscoring the possible influence of environmental, climatic, and management factors specific to each region. The high prevalence in Africa raises concerns about potential zoonotic transmission and the implications for both animal and human health, necessitating further investigation into the reservoirs and transmission dynamics within this continent. The prevalence by WHO region showed that the AFR region had the highest rate at 72.3% (95% CI: 61.7%–80.8%, one study), while the EUR region had the lowest at 6.9% (95% CI: 2.1%–20.4%). This variability underscores the influence of environmental factors, management practices and population density on the prevalence of this parasite. Country‐based prevalence analysis revealed that the highest *G. duodenalis* rates in rabbits were 100% in the UAE, 72.3% in Nigeria, 40% in Brazil and 27.8% in Spain. Conversely, the lowest rates occurred in Russia (1.8%), Australia (2.3%), the USA (3.1%) and Croatia (3.4%). These findings highlight significant geographic disparities in the prevalence of *G. duodenalis* among rabbit populations. Notably, the high prevalence observed in these countries may be attributed to environmental factors, husbandry practices or potential contact with other infected animals. On the other hand, the markedly low rates in Russia, Australia, the USA and Croatia raise questions regarding the effectiveness of biosecurity measures and parasite management strategies in these regions. These countries may benefit from enhanced surveillance and control programs to further mitigate the spread of parasitic infections such as *G. duodenalis* among their rabbit populations. Sample size–based prevalence indicated a direct correlation between larger study samples and a reduced prevalence of *G. duodenalis* in rabbits. This observation suggests that the estimator's accuracy improves with increased sample sizes, allowing for a more precise representation of the *G. duodenalis* infection rates within rabbit populations. Utilizing larger cohorts in future studies may not only enhance the reliability of prevalence estimates but also contribute to a more comprehensive understanding of the epidemiological patterns associated with *G. duodenalis* infestations. In turn, this knowledge could inform effective management strategies and policies aimed at controlling the spread of this parasite in both wild and domesticated rabbit populations. Of note, most aforementioned results are derived from available information and individual studies, so this study's interpretation is based on that data. Expanding research across different geographical areas could significantly alter the findings and current interpretation.

The molecular analysis uncovered significant genetic diversity (assemblages A, B and E) within *G. duodenalis* isolates from rabbits, with assemblage B being the most frequently identified. This is in line with previous studies indicating that assemblage B is commonly associated with humans (Ryan and Cacciò 2013), suggesting potential zoonotic implications. The identification of sub‐assemblages AI and BIV warrants further investigation into their specific epidemiological roles and contributions to transmission dynamics. Sub‐assemblage AI was reported in both humans and animals, and sub‐assemblage BIV was reported in humans as well as companion and wild animals (Mbae et al. 2016; Pipiková et al. 2020). Of note, out of 19 molecular studies conducted, only 6 studies investigated *G. duodenalis* sub‐assemblages in rodents and those 6 studies did not evaluate all their positive samples. Therefore, it is expected that there are various sub‐assemblages of *G. duodenalis* in rabbits. Overall, understanding the genetic makeup of *G. duodenalis* will be crucial for addressing public health concerns, especially in mixed‐use environments where domestic and wild populations interact.

Among the most important limitations of the current study, we can point out the lack of sufficient studies from different geographical regions, the low sample size in the evaluated studies and the analysis based on single studies, which can affect the results.

## 5 Conclusion

This systematic review and meta‐analysis highlighted a notable global prevalence of *G. duodenalis* in rabbits (12.1%), characterized by significant genetic diversity and potential zoonotic implications (assemblages A, B and E/sub‐assemblages AI and BIV). The findings underscore the necessity for continued surveillance and research on *G. duodenalis* in rabbit populations to inform public health policies and improve management practices aimed at mitigating zoonotic transmission.

## Author Contributions

**Ali Asghari**: conceptualization, methodology, software, data curation, investigation, writing–original draft, writing–review and editing. **Mohammad Reza Mohammadi**: investigation, writing–review and editing, methodology, writing–original draft. **Leila Naseri**: investigation, writing–review and editing. **Laya Shamsi**: investigation, methodology, writing–review and editing. **Milad Badri**: investigation, methodology, writing–review and editing. **Ali Pouryousef**: conceptualization, investigation, writing–original draft, writing–review and editing.

## Conflicts of Interest

The authors declare no conflicts of interest.

### Peer Review

The peer review history for this article is available at https://publons.com/publon/10.1002/vms3.70176

## Supporting information



Supporting Information

Supporting Information

Supporting Information

Supporting Information

Supporting Information

Supporting Information

## Data Availability

The datasets used and/or analysed during the current study are available in the online version.
