# Supplementary material for: A Global Systematic Review and Meta‐Analysis of Giardia duodenalis in Rabbits: Epidemiology, Genetic Diversity and Possible Zoonotic Concerns
Source: Vet Med Sci. 2024 Dec 18;11(1):e70176. doi: 10.1002/vms3.70176 (PMC11653087; doi:10.1002/vms3.70176)
Supplement: Supplementary file 1 — Supporting Information [file VMS3-11-e70176-s001.docx]

**Supplementary Fig. 1.** The global prevalence of *G. duodenalis* in rabbits based on publication years.
