# Supplementary material for: A Global Systematic Review and Meta‐Analysis of Giardia duodenalis in Rabbits: Epidemiology, Genetic Diversity and Possible Zoonotic Concerns
Source: Vet Med Sci. 2024 Dec 18;11(1):e70176. doi: 10.1002/vms3.70176 (PMC11653087; doi:10.1002/vms3.70176)
Supplement: Supplementary file 6 — Supporting Information [file VMS3-11-e70176-s002.docx]

**Supplementary Table 1**

**JBI critical appraisal checklist applied for included studies**

| Author Name/Year | Sample was representative? | Participants appropriately recruited? | Sample size was adequate? | Study subjects and the setting described? | Data analysis conducted | Objective, standard criteria, reliably used? | Appropriate statistical analysis used | Confounding factors/ subgroups/ differences identified and accounted? | Subpopulations identified using objective criteria | Overall quality |
| --- | --- | --- | --- | --- | --- | --- | --- | --- | --- | --- |
| Pacha, 1987 | Yes | Yes | Yes | Yes | Yes | Yes | No | No | No | 4/9 |
| Chilvers, 1998 | Yes | No | Yes | Yes | No | Yes | No | No | No | 6/9 |
| Sulaiman, 2003 | Yes | No | Yes | No | Yes | No | Yes | No | No | 4/9 |
| Lebbad, 2010 | Yes | Yes | No | Yes | No | Yes | No | Yes | No | 5/9 |
| Beck, 2011a | Yes | Yes | Yes | Yes | Yes | No | Yes | Yes | No | 7/9 |
| Beck, 2011b | Yes | Yes | Yes | Yes | Yes | No | Yes | No | No | 6/9 |
| Zhang, 2012 | Yes | Yes | Yes | Yes | Yes | No | Yes | Yes | Yes | 8/9 |
| Nolan, 2013 | Yes | Yes | Yes | Yes | Yes | Yes | Yes | Yes | No | 8/9 |
| Rewatkar, 2013 | Yes | Yes | Yes | Yes | Yes | No | Yes | No | Yes | 7/9 |
| Liu, 2014 | Yes | Yes | Yes | Yes | Yes | No | Yes | Yes | Yes | 8/9 |
| Pantchev, 2014 | Yes | No | Yes | Yes | Yes | Yes | Yes | No | Yes | 7/9 |
| Qi, 2015 | Yes | Yes | Yes | Yes | Yes | Yes | No | No | No | 4/9 |
| Koehler, 2016 | Yes | No | Yes | Yes | No | Yes | No | No | No | 6/9 |
| Mosallanejad, 2017 | Yes | No | Yes | No | Yes | No | Yes | No | No | 4/9 |
| Akinkuotu, 2018 | Yes | Yes | No | Yes | No | Yes | No | Yes | No | 5/9 |
| Jiang, 2018 | Yes | Yes | Yes | Yes | Yes | No | Yes | Yes | No | 7/9 |
| Marhoon, 2018 | Yes | Yes | Yes | Yes | Yes | No | Yes | No | No | 6/9 |
| Sarzosa, 2018 | Yes | Yes | Yes | Yes | Yes | No | Yes | Yes | Yes | 8/9 |
| Zhang, 2018 | Yes | Yes | Yes | Yes | Yes | Yes | No | No | No | 4/9 |
| Kurnosova, 2019 | Yes | No | Yes | Yes | No | Yes | No | No | No | 6/9 |
| Li, 2020 | Yes | Yes | Yes | Yes | Yes | No | Yes | Yes | No | 7/9 |
| Zahedi, 2020 | Yes | Yes | No | Yes | No | Yes | No | Yes | No | 5/9 |
| Elbakri, 2021 | Yes | Yes | Yes | Yes | Yes | Yes | No | No | No | 4/9 |
| Tang, 2021 | Yes | No | Yes | Yes | No | Yes | No | No | No | 6/9 |
| Baptista, 2023 | Yes | Yes | Yes | Yes | Yes | No | Yes | No | No | 7/9 |
| Rego, 2023 | Yes | Yes | No | Yes | No | Yes | No | Yes | No | 5/9 |
